# Diagnosis and Pulp Treatment

**Published:** 1911-11-15

**Authors:** G. L. Powers

**Affiliations:** Paris, Tenn.


### DIAGNOSIS AND PULP TREATMENT.

BY G. L. POWERS, D.D.S., PARIS, TENN.

Read before the Tennessee State Dental Association, May 25, 1911.

Mr. President and members of the Tennessee Dental Association: In appearing before you with the subject I have chosen, let it be understood that I disclaim any pretention of having any thing new, or unusual. This shall be a reading concerning a subject in which I am interested, and one which I feel is of great importance to the profession. The ideas expressed in this paper may not coincide with your ideas of treating dental pulps, but they may serve as a medium through which more good ideas and methods of treatment may be brought out in subsequent discussion.

The subject of diagnosis and pulp treatment has not received the attention it should, at least I feel this to be true in some quarters. The manner in which some of these troubles are treated is unwarranted and inexcusable. Knowing the action of the medicines we use and the effect these medicines have when applied to the pathological conditions we are treating is of vital importance. The intelligent professional man in dentistry can not be satisfied with using medicines which happen to possess certain antiseptic qualities and which after an inexcusable length of time might finally relieve the case, but he should know why he uses certain medicines, what their actions may be and what results may be expected from their use. We occasionally see some few men in the profession who only rely upon a very few antiseptic drugs using them on all occasions, regardless of conditions.

I knew of a simple case that was treated for about a year without results. The patient went from time to time for treatment and each time the dentist would remove the cotton, detect the odor and say that the tooth would have to be treated again, or until there was no odor. The treatments were never sealed in the tooth and of course the cotton and medicine was always foul and had an odor. The dentist lost the patient. Patient applied to another dentist and tooth was filled after the third visit. I mention this to emphasize the difference in accurate and inaccurate treatment.

Of course we do not all treat teeth in the same manner. That would destroy our individuality. What would be success in one man's hands might be a failure in anothers, but let us strive for definite results as intelligently as possible.

Before we can put into service any drug or drugs, we must first have an intelligent ability to diagnose diseases. Failure to differentiate these matters means failure to all efforts. In this discussion we have to consider the dental pulp as to its anatomy, physiology, pathology, differential diagnosis and treatments.

In my opinion, we are seldom, if ever, excusable for not hermetically sealing all remedies into the teeth. Many will differ in opinion on this, but if you do not seal the case you can not get an intelligent idea of the conditions as they actually exist in the pulp canals unhampered by the secretions of the mouth and foreign matters which may accumulate. When the tooth is not sealed the treatment is more or less unreliable. If the remedies are left in the teeth on cotton it soon gets vitiated and the canals necessarily get foul and you cannot tell whether the odor or secretion is from the root or from extraneous contamination.

Before we can diagnose a case we must have a considerable knowledge of the anatomy, physiology and pathology of the part. Then too, before we can apply remedies, which will be specific we must have a knowledge of the action of drugs. To be able to seal in our remedies, we must know diagnosis and so understand the action of the remedies we propose to use that we may not make a mistake and stir up more trouble than we already had present.

We know that a perfectly healthy pulp is not severely sensitive to the ordinary irritants, and the patient has perfect comfort while it performs its functions. When perfectly normal, it is so non-responsive that in many cases the pulp may be punctured painlessly.

The pulp is composed of connective tissue, of a soft variety, and is well supplied with arteries, veins and nerves, as the main portion of its structure. The arteries enter the pulp at the apical end of the tooth, as branches of the larger arteries and divide into capillaries which are profusely distributed throughout the pulp. The veins form a similar net work of vessels and increase in size as they approach the apical portion of the pulp, and make exit through the apical foramin to enter the larger veins. They receive, when in normal state, all the blood which is brought into the pulp by the arteries. The nerves of the pulp enter through the apical foramin and are widely distributed through the pulp tissue. The walls of the vessels of the pulp are thin and differ somewhat from the vessels in other portions of the body, except the brain. Unlike other parts of the body, except the brain, the pulp tissue has no lymphatics. So, considering the fact that it is confined within the bony walls of the tooth, the vessels tender and that it is supplied with no lymphatics, and consequently subject to so many disturbances makes the dental pulp demand considerable attention. Such delicate tissue will respond to irritants readily and if the irritation is continued, the pulp tissue will experience the various stages of inflammation and finally die.

*Pulp Pathology.* A great many teeth come under our observation presenting conditions which makes it difficult for us to diagnose, or rather to know just what is for the best interest of the patient. Whether to save the pulp or destroy it. It is so easy, in many cases to destroy and remove pulps that too many times we do not exercise enough conservatism in saving them. When we think, though, of the fine, tortuous canals in some of the teeth, the temporaments of some of the patients, the difficulty of removing all the dead tissue from the canals and the liability to fail to fill the canals so they will remain permanently aseptic, it is enough to cause us to stop and think it over. This is not an appeal for the too conservative retention, but rather one to prevent the too erratic destruction of pulps.

*Hyperemia.* In considering the inflammatory process of the pulp I wish to divide it into three stages, namely, hyperemia, congestion and stasis.

As a result of some irritant, we have as a reaction, through the agency of its nervous system, an increased amount of blood to the part. The arteries are slightly changed as result of the hyperemia. They are slightly distended and walls of the capillaries are relaxed and nutrition is disturbed.

*Congestion:* If the irritation is kept up or increased, as in caries, the blood supply to the pulp is still more increased, more red blood corpuscles rush in until the vessels are congested and dilated, more blood has come into the part than gets away. The blood current slows, more red and white corpuscles come, the red ones on the center and the white ones on the outer portion of the current, next to the walls of the vessel.

*Stasis.* At this stage of the inflammation we have stagnation of the blood flow, diapedesis and exudation of serum through the walls of the vessels. Pain, worse, longer history, and general conditions advise us pulp is about dead. This serum from the blood is highly coagulable and when, after passing from the vessel into the effected tissue it is coagulated, the pulp having no lymphatics, it is destined to disintegrate and die. There are present none of the agencies which effect resolution.

Too many times only enough information is obtained to enable the operator to tell whether the pulp is dead or alive. If it is a patient with an aching tooth shall we apply arsenic, or cocain, and settle the matter? Sometimes too, when a tooth is opened into and found to contain putrescent canals, it is left open and dressed with some convenient remedy, many times contraindicated. Many teeth on being opened into are found to have pus in the pulp chamber and at the same time to have living apical portions. So, it is important to see whether the pulp is in a state of hyperemia, congestion, stasis, death, putrescent canals with gaseous matters involved, or in a septic-vital state. Therefore when a patient presents with an aching tooth it is our duty not only to see whether the pulp is dead or alive, but to find out the exact stage of its pathology.

### DIFFERENTIAL DIAGNOSIS.

*Hyperemia.* When patient comes with acute hyperemia, the symptoms will be slight disturbance by pain due to some irritant, only on the application of the irritant. Pain subsides on its removal.

*Treatment.* Remove the irritant, or cause. If the trouble is caused by caries, wash out the cavity with warm antiseptic solution and apply anodyne-aseptic treatment, such as phenol fluid, one ounce and thymol grs. 48. This relieves the pain and sterilizes the underlying septic dentine. Care should be exercised and not seal the remedy in with pressure if there is actual exposure, as this might irritate the pulp and cause the very trouble we are trying to abort. Soft cement is preferable for sealing in the medicine. After this has been treated once, if the case presents a favorable history, the remaining decayed dentine may be removed and the tooth filled.

*Congested Pulp.* In congestion we have much more blood to the pulp than we had in hyperemia and the pain is more intense and does not stop of its own accord. The pain comes on spontaneously, without the aid of an irritant, and is sometimes of a jumping nature, worse in excitement and when lying down. Irritants increase the pain and it continues after the irritant has been removed.

*Treatment.* After removing as much of the decayed dentine as is practicable, wash out cavity with warm solution and place the phenol-thymol solution referred to above, sealing it into the cavity. Counter irritate the gum with equal parts aconite and iodine. If the case is of sufficient severity, hot foot baths, for thirty minutes before retiring, is good. If the system is constipated and disorder prevails, may use laxatives or cathartics.

*Stasis.* In this stage warm applications irritate, cold relieves, pain intense, the pulp must be destroyed either by anesthetizing or devitalizing by arsenic. If the cavity is accessible and the pulp is not too highly inflamed, the cavity may be excluded from moisture, the dentine sterilized with thymol or 1-500 bi-chloride solution, most of the decay removed and the pulp anesthetized by pressure method and removed. It is necessary to sterilize the underlying dentine in order to prevent the forcing through the apical foramin of poisonous matters. We can not always determine the limit of the cocain anesthesia. It necessarily at times goes beyond the end of the root. If the solution is septic we infect the pericementum and surrounding tissues and set up pericementitis. The pulp may be removed after the hemorrhage is over the canal may be cleansed and filled immediately, or the canal may be sealed with a dressing of pure creosote and filled at the next sitting

*Devitalization.* When the tooth is devitalized by the use of arsenic it is not necessary to sterilize the dentine as in the anesthesia method, for the decayed matter is not forced into the pulp and apical end tissues and it can be removed after the pulp is devitalized painlessly. Let the arsenic stay in tooth 24 to 48 hours. I prefer a paste composed of arsenic grs. 10, cocain grs. 20, and carbolic acid to make a stiff paste. Seal this hermetically into the cavity, being very careful about the cervical margin. Never, under any circumstances, make an exposure of the pulp previous to the application of the arsenical paste, as it is too cruel to the patient, and is absolutely unnecessary. When the patient returns, second visit, the bulbous portion, or all, the pulp may be removed and the root filled immediately, or it may be dressed with pure beech-wood creosote and have the patient return later to have the root filled. These are matters at the discretion of the operator.

If desired, the bulbous portion of the pulp at the second sitting may be removed with a suitable round bur and that portion of the tooth filled with a thick paste composed of para-formaldehyde dram 1, oxide of zinc, dram 2, thymol dram 1, and glycerine to make a stiff paste. This sealed with soft cement, without pressure. Later the tooth may be filled as usual. If it is necessary the whole tooth may be finished at the second sitting. Some will shake their heads at mummification, but if you will use this paste properly you will have indeed very pleasant results, both to yourself and patient. I was just as conservative as any one on this mummifying process, but in four years of occasional use I have had satisfaction. You will have no necessity for shaking your head if the operation is done properly.

*Septic-vital Pulps.* Many times, on opening into cavities, we find the tooth evolves pus, but after the pus is removed, we find the apical portions of the pulp is alive and sensitive. Many others are entirely dead and evolve gases. It is important to distinguish the two pathological conditions.

In the first case, remove the pus, wash out and seal into the cavity, phenol and thymol. At the second visit if the surface is still sensitive, gently work a little aromatic sulphuric acid into the canals. By this method they can usually be removed painlessly. Sometimes it is necessary to use slight pressure anesthesia, before removing the remnants. After their removal, dress canals as usual and fill.

*Pulp Capping.* If the pulp is in a healthy condition, and exposed, a paste of a non-irritating-aseptic nature is permissible and in a great many cases advisable. Creosote, pure beech-wood, and the powder of your cement made into a paste and applied to the exposure, sealed in with soft cement is good. The paste should be spread over the pulp portion to prevent the irritating effect of the acid in the cement covering. If the pulp is not in a healthy condition it should not be capped. In fact capping should be done sparingly. There are many cases, such as lower first molars in childrens mouths where the roots are not fully developed, in which it is the thing indicated to do. Pulp capping is a failure when the pulp is in any of the advanced stages of inflammation.

*Putrescent Pulps.* These have a specific in formaldehyde—cresol treatment, matters which I referred to in a paper at Memphis, two years ago.

DISCUSSION.

DR. STANLEY RICH: I agree with the Doctor in practically all that he has said. There is one point, however, that I want to take issue with him on, and that is that we should always seal up these cases. I think there is a class of cases in which it is better to put in a treatment and seal it in loosely. It sometimes depends upon the drugs we use, whether they should be hermetically sealed or not. For example take a tooth which has reached that stage of inflammation or trouble that there remains a septic or putrescent pulp in the tooth, it is not advisable to open into that tooth, because there is a great deal of danger of forcing infection through the apical foramen, and it will require some time for it to penetrate that region. I think the proper treatment is to open the pulp chamber thoroughly without producing any pressure upon the contents and drain it out as thoroughly as possible, and then seal loosely with a cotton dressing. After waiting for one or two days dry it out by the same process and we may then treat it more perfectly, apply the rubber dam and remove practically all of the contents and apply the cresol and seal. With a tooth very sore to pressure this treatment is much more preferable to immediate sealing because it gives the patient much less pain and we get the same results though it will take a little longer.

Now there is one other point, and that is the capping of pulps. I think we have gone entirely too far in the destruction of the pulps of teeth. Some men say that we should never place a crown on a tooth without devitalizing it. They tell you that it is easier to treat it, but a devitalized tooth has not the strength that a vital tooth has, and they become very brittle, break and decay more readily. There is only one thing I wish to say and that is that there should be a disk of some sort placed in on the paste, or cement powder or zinc oxid to prevent pressure in putting in the cement later on.

Dr. Cattell: I did not have anything particular to say awhile ago, but now I have. There was a suggestion or two brought out later on that I cannot allow to go by unchallenged. Why do we begin treating an infected pulp? If there is a septic pulp, is it not to relieve the condition caused by micro-organic life? And yet at the first treatment it is proposed that we still continue conditions which allow micro-organic life to sweep into it by the million. Our disinfectant that we put there for the purpose of overcoming the infection should have a chance to operate without hindrance it should be isolated. It is the concensus of opinion of all bacteriologists that when we have a case of infection it is our business to stop it.

Another statement that was made in the previous discussion should be qualified I believe. The Doctor said that it was a well-known fact that the pulpless tooth becomes in time more brittle, loses a certain amount of integrity it is true, but he says that decay will come on more rapidly. Now what effect the pulpless tooth may have on the action of micro-organism I do not know, but a carefully sterilized pulpless tooth if properly filled ought not to decay readily.

Dr. Leonard: I would like to say a few words after congratulating the essayist, I think this is one of the very best essays that I have ever heard in the Association.

I wish to speak first on the point made by the last speaker as to the disinfecting agent being sealed into the cavity. I quite agree with Dr. Rich in that no matter how powerful the disinfecting agent, we know that there are gases evolved from the decomposing articles. We seal them in there and they are going to produce pressure and consequent irritation at the apical end which is apt to result in further inflammation. All the agents we use for disinfecting are not really soluble in the fluids of the mouth, they will remain constant and undiluted for a long period, for weeks without having been washed out and without any chance of infection coming from without and coming up to the point we are trying to protect. It is simply a dressing. Take for instance some of the essential oils. They are not soluble in the fluids of the mouth, and that would certainly protect the tooth from further infection from without.

As to pulp capping I agree with the essayist. I think only in selected cases should we ever use pulp capping. I think the danger of septic conditions arising from the death of the pulp is a much greater menace to the comfort of the patient, and the integrity of the tooth, that is to the usefulness of the tooth, than the removal of the septic pulp and rendering the canals sterile at that sitting.

Now as to the lymphatics in the pulp. I doubt if it is an accurate statement to say that there are no lymphatics in the pulp. It is true that it has not been proven that there are, but it is true on the other hand that it has not been proven that there are not, and there is some evidence that there are lymphatics, or something to take the place of lymphatics in the pulp. A gentleman was telling me yesterday of a tooth that he had injured somewhat in some orthodontic operations that had turned perfectly pink, in other words, he said that he had got a pink tooth, and that the pulp would soon die and he would have to cut off the tooth and put on a crown, but he was surprised to find this condition was gradually being taken off and the pink condition disappeared in a short while and it is now a perfectly healthy tooth. I have seen the tooth myself and know it to be a good tooth. I do not see how that condition could have been corrected unless there were lymphatics in the pulp and there is an absorption of material that was present and pulps that were up in the middle of the crown have receded until they were below the cervical margin. Certainly there is something that takes the place of lymphatics in the pulp chamber.

Dr. B. H. Johnson, of Portland, Tennessee: Speaking of my way of treating such teeth, instead of leaving the cavity entirely open, I seal in my treatment with gutta-percha, and then make a puncture with a small smooth broach that will leave an opening plenty large to allow the gases to escape while not admitting any moisture. I have found that a very successful way. That prevents any infection or moisture from getting in from without, and also keeps in the treatment very successfully.

Dr. Rich: Dr. Cattell says that in his treatment of these cases—he uses the word micro-organisms; I want to say that the patient does not come to you to have his micro-organisms assassinated; he comes to get relief, and I want also to say that the amount of pain a patient is suffering and will continue to suffer from probably twelve to twenty-four hours, will do that patient a great deal more physical injury than to allow those organisms to exist a little bit longer. He says he sees no reason for the tooth that has had the pulp removed to become brittle and increase the liability to decay. A tooth, after the pulp has been removed is more brittle and weaker in other respects and it becomes more susceptible to the action of bacteria. This is so obvious as to need no restatement.

Dr. Powers: I wish to thank the Association for the cordial reception of my paper, and wish only to refer to one or two matters. First the importance of sealing of the tooth. When a patient comes into my office with a dead tooth, a pulp chamber containing a putrescent condition, full of gases, but the pulp not exposed, I make an exposure, and simply clean out as much debris as I can and put in a solution of formaldehyde-cresol and seal it, without irritating the gum, and instead of increasing or continuing the pain, it will not be an hour until that pain is relieved. The next day the patient will come back with a tooth that is next to all right, if not all right, the soreness gone. I do not like the idea of dodging the issue, neither do I like the idea of puncturing a hole in the gutta-percha, cement or whatever it is. If you havn't enough confidence in the drugs you put in there, well you had better get some other kind of medicine.

